# Psychological distress and fear of Covid-19 in student nurses before clinical placement: a cross-sectional study

**DOI:** 10.1590/1980-220X-REEUSP-2021-0548en

**Published:** 2022-05-20

**Authors:** Ana Belén Subirón-Valera, Ana Carmen Lucha-López, Beatriz Rodríguez-Roca, Fernando Urcola-Pardo, Ana Anguas-Gracia, Pedro José Satústegui-Dorda, María Teresa Fernández-Rodrigo, Isabel Antón-Solanas

**Affiliations:** 1 Universidad de Zaragoza, Facultad de Ciencias de la Salud, Departamento de Fisiatría y Enfermería, Zaragoza, Spain.; 2 Universidad de Zaragoza, Instituto Universitario de Investigación en Ciencias Ambientales de Aragón, Grupo de investigación Agua y Salud Ambiental, Zaragoza, Spain.; 3 Instituto de Investigación de Aragón, Grupo de investigación en Cuidados, Zaragoza, Spain.; 4 Instituto de Investigación de Aragón, Grupo de investigación Seguridad y Cuidados, Zaragoza, Spain.; 5 Instituto de Investigación de Aragón, Grupo de Investigación en Enfermería en Atención Primaria en Aragón, Zaragoza, Spain.

**Keywords:** Students, Nursing, COVID-19, Professional training, Mental Health, Anxiety, Psychological Distress, Estudantes de Enfermagem, COVID-19, Capacitação Profissional, Saúde Mental, Ansiedade, Angústia Psicológica, Estudiantes de Enfermería, COVID-19, Capacitación profesional, Salud Mental, Ansiedad, Distrés psicológico

## Abstract

**Objective:**

To determine the degree of psychological distress and fear of COVID-19 experienced by undergraduate student nurses who were about to begin their clinical placements.

**Method:**

A cross-sectional study was carried out with 100 second- and third-year undergraduate student nurses of the University of Zaragoza (Spain). Measures included the Fear of COVID-19 Scale and the Depression Anxiety Stress Scales.

**Results:**

Regularly, student nurses did not think of themselves as vulnerable to COVID-19. However, a significant association was observed between the student nurses’ level of psychological distress and cohabiting with relatives or people who were considered vulnerable to the infection (p = 0.035). The Depression Anxiety Stress Scale results revealed a low level of psychological distress in general; the Fear of COVID-19 Scale indicated moderate fear (2.94).

**Conclusion:**

Student nurses who lived with their relatives experienced higher levels of stress due to the perceived risk of transmission, but were less fearful of loss of work and income. Anxiety in our sample was associated principally with not knowing their upcoming placement location.

## RESUMEN

**Objetivo:**

Determinar el grado de angustia psicológica y miedo al COVID-19 que experimentan los estudiantes de pregrado de enfermería que estaban a punto de empezar sus prácticas clínicas.

**Método:**

Se realizó un estudio transversal en 100 estudiantes de enfermería de segundo y tercer año de la Universidad de Zaragoza (España). Las medidas incluyeron la Escala de Miedo a la COVID-19 y la Escala de Depresión, Ansiedad y Estrés.

**Resultados:**

Normalmente, los estudiantes de enfermería no se consideraban vulnerables a la COVID-19. Sin embargo, se observó una asociación significativa entre el nivel de distrés psicológico de los estudiantes de enfermería y la convivencia con familiares o personas percibidas como vulnerables a la infección (p = 0,035). Los resultados del Escala de Depresión, Ansiedad y Estrés revelaron un bajo nivel de distrés psicológico en general; la Escala de Miedo a la COVID-19 indicó un miedo moderado (2,94).

**Conclusión:**

Los estudiantes de enfermería que vivían con sus familiares experimentaron mayores niveles de estrés debido al riesgo percibido de transmisión, pero tenían menos miedo a la pérdida de trabajo e ingresos. La ansiedad en nuestra muestra se asoció principalmente con el desconocimiento de su próxima ubicación de colocación.

## RESUMO

**Objetivo:**

Determinar o grau de sofrimento psicológico e medo da COVID-19 experimentado por estudantes de enfermagem que estavam prestes a iniciar seus estágios clínicos.

**Método:**

Um estudo transversal foi realizado em 100 estudantes de enfermagem do segundo e terceiro anos da Universidade de Zaragoza (Espanha). As medidas incluíram a Escala de Medo da COVID-19 e as escalas de Depressão, Ansiedade e Estresse.

**Resultados:**

Regularmente, os estudantes de enfermagem não se consideravam vulneráveis à COVID-19. No entanto, observou-se associação significativa entre o nível de sofrimento psicológico dos estudantes de enfermagem e a convivência com familiares ou pessoas percebidas como vulneráveis à infecção (p = 0,035). Os resultados da Escala de Depressão, Ansiedade e Estresse revelaram um baixo nível de sofrimento psicológico em geral; a Escala de Medo da COVID-19 indicou um medo moderado (2,94).

**Conclusão:**

Estudantes de enfermagem que moravam com seus familiares experimentaram níveis mais altos de estresse devido ao risco percebido de transmissão, mas tinham menos medo de perda de trabalho e renda. A ansiedade em nossa amostra foi associada principalmente ao desconhecimento de seu próximo local de estágio.

## INTRODUCTION

The 2019 novel coronavirus disease (COVID-19) pandemic has had a worldwide impact, causing dramatic loss of human life. The emergence and unprecedented spread of COVID-19 has posed substantial challenges to the practices of everyday life^(1)^, including university teaching and learning^(2)^. In Spain, a nationwide lockdown was officially announced on 15^th^ March, 2020, to control the spread of the virus. This resulted in the suspension of classroom teaching and subsequent adaptation (with more or less success) to e-learning. In addition, clinical placements were suspended for undergraduate healthcare students^(3)^, raising concerns regarding the trainees’ clinical skills competence and progression.

University students are susceptible to experience a variable degree of psychological distress, including symptoms of anxiety, depression, and stress^(4)^. Common sources of psychological distress in this population are related to academic and psychosocial pressures^(5)^, including heavy workloads, assessment tasks, insufficient leisure time, competitiveness, meeting parents’ expectations, forming and maintaining new relationships and, often, moving to a new place. Other risk factors for psychological distress include age and sex, with women being more vulnerable than men^(6)^, and financial pressures^(7)^. Healthcare students experience a moderate level of psychological distress^(8)^, but student nurses, in particular, experience a higher degree of anxiety in relation to their clinical placements^(9)^.

Spain is no longer under lockdown, but restrictions to public mobility and social distancing measures are still in force. Such measures continue to affect academic programs, educational approaches and methodologies, teaching and learning activities and assessment tasks. This has resulted in an unprecedented degree of uncertainty, which has affected students and teachers’ lives alike^(10)^. Uncertainty is probably the most significant consequence of the pandemic for higher education staff and students^(11)^. The degree of uncertainty experienced by nurse students and teachers alike at the beginning of the academic year 2020-2021 was very high. In the case of the student nurses who were about to begin their clinical placements after summer break, this state of uncertainty was aggravated by the fact that they were about to access clinical areas, thus being in close contact with diagnosed and undiagnosed cases of COVID-19.

There is growing concern for well-being of future healthcare professionals in the midst of the COVID-19 pandemic and it may have an impact on their academic progression, skills competence, and professional commitment^(12)^. The COVID-19 pandemic is affecting population’s general health^(13)^, in particular women, with student and qualified nurses being the most affected. This increased level of psychological distress is due to close contact with the virus during placements and shifts, the fear of being infected and/or infecting others, such as close friends, relatives, cohabitant peers or vulnerable people in general. Specifically, in the case of students, the fear of academic delay and/or missed opportunities for learning could be added. Furthermore, nursing students may lack information, or have misconceptions, about the disease and infection control measures relevant to clinical practice^(14)^, which may result in increased infection transmission and could increase the students’ psychological distress^(15)^.

The aim of this research was to determine the level of psychological distress and fear of COVID-19 in a sample of undergraduate student nurses who were about to begin their clinical placements during the academic year 2020-2021. In addition, we analysed the association between the students’ level of psychological distress and fear of COVID for themselves and for their cohabiting relatives and/or significant others.

## METHOD

### Design

Cross-sectional study of psychological distress and fear of COVID-19 in a sample of undergraduate student nurses who were about to begin their clinical placements during the academic year 2020-2021. The manuscript was written in accordance with the Strengthening the reporting of Observational Studies in Epidemiology (STROBE) protocol^(16)^.

### Population and Local

Following the universal sampling yardstick, all the second- and third-year undergraduate student nurses were informed about the project and were invited to participate voluntarily if they complied with inclusion and exclusion criteria. A total of 100 undergraduate student nurses registered in the bachelor’s degree in nursing offered by the University of Zaragoza during the academic year 2020-21 took part in this study. Participant recruitment and data collection took place from October to November 2020. The students were informed about the aims of the study and the methods of data collection by a researcher in the classroom; a copy of the participant information leaflet and consent form was made available to the students at this time. The students were assured that privacy and confidentiality would be maintained, and that they had the right to refuse to participate in the study or to withdraw consent to participate at any time without reprisal.

Inclusion criteria included: 1) being registered in the bachelor’s degree in nursing at the University of Zaragoza and 2) giving their informed consent to participate in the study. We excluded students who: 1) had already commenced their clinical placements at the time of data collection, and 2) had a formal diagnosis of mental illness. First year students were excluded as they do not undertake clinical placements during the first academic year, and fourth year students were also excluded as they had already commenced their clinical placements at the time of data collection.

### Data Collection and Study Procedures

Data were collected using a self-administered, anonymous electronic survey designed through Google Forms and accessible only to second- and third-year student nurses through the institutional learning platform Moodle. Three lecturers and co-authors participated in the data collection process. Data were collected in the classroom at the beginning of the teaching sessions after explaining the study aims and objectives, and guaranteeing anonymity and confidentiality. In addition, the students were assured that participation was voluntary and that no consequences would follow from a decision not to participate. All the participants were informed about the nature of the study, the objectives, and that they could abandon the study if they wanted without giving any explanations. Consent to participate in the study was implied when the student nurses voluntarily completed and sent the electronic survey.

A questionnaire of sociodemographic variables was designed ad hoc in order to describe the characteristics of our sample, including age, sex, living arrangements, perception of personal vulnerability to COVID-19, perception of cohabitants’ vulnerability to COVID-19, clinical placement location, year of study, and prior clinical experience.

The Fear of COVID Scale and DASS-21 Scale were used in their already translated Spanish version in both cases. The student nurses’ fear of COVID-19 was assessed using the Fear of COVID-19 Scale (FCS), designed and validated in the general Spanish population^(17)^. This scale comprises 18 items measuring the intensity of fears and concerns in relation to COVID-19 and is subdivided into 4 dimensions, namely fear of infection, illness and death, fear of lack of resources, fear of social isolation, fear of loss of employment and income. The instrument is answered on a 5-point intensity scale ranging from 1 (not at all or very little) to 5 (very much or extremely). Cronbach’s alpha for the total scale was 0.89.

The level of psychological distress in our sample was measured through the Depression Anxiety Stress Scales (DASS-21) in its Spanish version (18). The DASS-21 is a 21-item scale comprising three subscales: DASS-D (depression), DASS-A (anxiety) and DASS-S (stress). Respondents must rate the extent to which each statement applies during the past week on a 4-point Likert scale ranging from 0 (did not apply to me at all) to 3 (applied to me very much). Because the DASS-21 scale is a short-form version of the DASS (42 items), the final score for each sub-scale is multiplied by two and evaluated according to its severity rating index. Depression, anxiety and stress scores are calculated by adding up the scores of the items in each separate subscale. The results are interpreted as follows: DASS-A (>19 = extremely severe anxiety; 19–15 = severe anxiety; 14–10 = moderate anxiety; 9–8 = mild anxiety; 7–0 = no anxiety/ normal), DASS-D (>27 = extremely severe depression; 27–21 = severe depression; 20–14 = moderate depression; 13–10 = mild depression; 9–0 = no depression/normal), DASS-S (>33 = extremely severe stress; 33–26 = severe stress; 25–19 = moderate stress; 18–15 = mild stress; 14–0 = no stress/normal).

The Spanish version of the DASS-21 scale has good psychometric properties with good reliability and validity; Cronbach’s alpha for the whole scale is 0,81, ranging from 0,73 to 0,81 for each separate dimension^(18)^.

### Data Analysis

Mean and standard deviation were used to represent continuous variables; frequencies and percentages were used to represent categorical variables. Normal distribution of the variables was verified through the Kolmogorov-Smirnov test. We used the Mann-Whitney test to perform the bivariate analyses, whereas multivariate inference was analyzed using the Kruskal Wallis test. The relationship between qualitative variables was analyzed using the Chi-square test. Data codification, processing and analysis were completed using the software Statistical Package for the Social Science (SPSS version 26 for Windows, IBM Corp., Chicago, IL, USA), accepting a level of significance of p < 0.05.

### Ethical Considerations

This study was reviewed and approved by the Clinical Research Ethics Committee of Aragón (Institute of Research of Aragón, Zaragoza) (IRB Ref: C.P. - C.I. PI20/499) prior to the start of this investigation. We confirm that each and every national and international standard for ethical research with human subjects was followed.

## RESULTS

The participants’ sociodemographic characteristics have been described in Table 1. Most of the participants were female student nurses (92%), and their mean age was 21.09 years. Approximately half of the participants were on the second year of studies (45%) and the other half were on their third year of study (55%). At the beginning of their clinical placement, most of the student nurses were living with relatives (83%). The vast majority of the participants did not have prior clinical work experience (90%) and did not consider themselves to be particularly vulnerable to COVID-19 (96%). Interestingly, one third of the participants did consider one or more of their cohabitants to be particularly vulnerable to COVID-19. Only 46% of the student nurses knew the location of their upcoming clinical placement at the time of entering this study.

**Table 1.** Description of the sociodemographic characteristics of the sample – Zaragoza, Spain, 2020.

Variables	N	%
**Sex**		
Female	92	92%
Male	8	8%
**Living arrangements**		
Alone	1	1%
Relatives	83	83%
Shared house/apartment	12	12%
Halls of residence	4	4%
**Perception of vulnerability of cohabitants to COV-19**		
No	66	66%
Yes	34	34%
**Perception of individual vulnerability to COV-19**		
No	96	96%
Yes	4	4%
**Year of study**		
2nd year	45	45%
3rd year	55	55%
**Clinical work experience**		
No	90	90%
Yes	10	10%
**Clinical placement location**		
Unknown	46	46%
Special service (ICU, Emergency, Surgery room)	27	27%
Hospital Ward	12	12%
Community care	15	15%

The results from the DASS-21 reveal that, in general, the student nurses’ level of psychological distress was low before commencing their clinical placements. However, the average score of the global FCS was 2,94, indicating a moderate fear of COVID-19 in our population, especially in relation to fear of infection illness and death (3,09), and fear of social isolation (3,11) (Table 2).

**Table 2.** Average scores from the DASS-21 and FCS scales – Zaragoza, Spain, 2020.

Dimensions and global scales	Mean (SD)	Range
DASS – Stress	13.62 (8.65)	0–38
DASS – Anxiety	7.26 (6.56)	0–26
DASS – Depression	8.66 (8.61)	0–34
DASS – Global	29.54 (20.81)	2–84
FCS – Fear of infection, illness and death	3.09 (0.67)	1.33–4.56
FCS – Fear of lack of resources	2.47 (1.00)	1–5
FCS – Fear of social isolation	3.11 (0.81)	1–5
FCS – Fear of loss of employment and income	2.76 (1.09)	1–5
FCS – Global	2.94 (0.66)	1.39–4.33

We dichotomized the variable living arrangements into living with relatives and other living arrangements, including living alone, sharing a flat or a house with peers, and living in halls of residence. We found that, generally, the student nurses’ level of psychological distress and fear of COVID-19 was slightly higher when they were living with their relatives. A significant difference was found in terms of fear of loss of employment and income between the students who were living with their relatives and those who lived alone or with peers (p = 0,026) (Table 3).

**Table 3.** Differences between psychological distress and fear of COVID-19 scores by living arrangement – Zaragoza, Spain, 2020.

Dimensions and global scales	Living arrangements	*n*	*Mean*	*SD*	*Z*	*p*
DASS – Stress	With relatives	83	13.66	9.02	0.212	0.832
	Other	17	13.41	6.77		
DASS – Anxiety	With relatives	83	7.88	6.89	1.677	0.094
	Other	17	4.24	3.38		
DASS – Depression	With relatives	83	8.87	8.71	0.398	0.690
	Other	17	7.65	8.28		
DASS – Global	With relatives	83	30.41	21.85	0.524	0.601
	Other	17	25.29	14.42		
FCS – Fear of infection, illness and death	With relatives	83	3.05	0.65	1.269	0.204
	Other	17	3.30	0.72		
FCS – Fear of lack of resources	With relatives	83	2.40	0.97	1.566	0.117
	Other	17	2.84	1.08		
FCS – Fear of social isolation	With relatives	83	3.10	0.82	0.056	0.956
	Other	17	3.14	0.80		
FCS – Fear of loss of employment and income	With relatives	83	2.64	1.03	2.229	0,026
	Other	17	3.35	1.21		
FCS – Global	With relatives	83	2.88	0.63	1.556	0.120
	Other	17	3.21	0.76		

A significant association was observed between the student nurses’ stress level specifically (p = 0.013), as well as their symptoms of distress (p = 0,035), and cohabiting with people who were perceived as being vulnerable to COVID-19 (Table 4).

**Table 4.** Differences between psychological distress and fear of COVID-19 scores by perception of vulnerability of cohabitants to COVID-19 – Zaragoza, Spain, 2020.

	Vulnerability of cohabitants	*n*	*Mean*	*SD*	*Z*	*p*
DASS – Stress	No	66	12.24	8.74	2.482	0,013
	Yes	34	16.29	7.94		
DASS – Anxiety	No	66	6.55	6.31	1.678	0.093
	Yes	34	8.65	6.91		
DASS – Depression	No	66	7.85	8.31	1.642	0.101
	Yes	34	10.24	9.08		
DASS – Global	No	66	26.64	20.27	2.109	0,035
	Yes	34	35.18	20.98		
FCS – Fear of infection, illness and death	No	66	3.05	0.64	0.992	0.321
	Yes	34	3.19	0.71		
FCS – Fear of lack of resources	No	66	2.40	0.99	1.025	0.305
	Yes	34	2.62	1.01		
FCS – Fear of social isolation	No	66	3.14	0.74	0.393	0.695
	Yes	34	3.04	0.95		
FCS – Fear of loss of employment and income	No	66	2.70	1.11	0.716	0.474
	Yes	34	2.87	1.06		
FCS – Global	No	66	2.90	0.63	0.754	0.451
	Yes	34	3.01	0.72		

We analyzed the student nurses’ level of psychological distress and their fear of COVID-19 in relation to the information received about their upcoming placement location. As it can be observed, those students who did not know their placement location experienced higher levels of anxiety (p = 0.029) and a greater fear of social isolation (p = 0.037) (Table 5). We analyzed other variables, namely perception of individual vulnerability to COVID-19 and year of study, in relation to the level of fear of COVID-19 and psychological distress, but we found no significant associations between them.

**Table 5.** Comparison of psychological distress and fear of COVID-19 scores between the students who knew their next placement location and those who did not know – Zaragoza, Spain, 2020.

	Categories	*n (%)*	Unknown (*n*= 46)	Known (*n*= 54)	*p*
DASS-S	No stress	62 (62%)	27 (58.7%)	35 (64.8%)	0.140 ^a^
	Mild	10 (10%)	6 (13%)	4 (7.4%)	
	Moderate	15 (15%)	4 (8.7%)	11 (20.4%)	
	Severe	11 (11%)	7 (15.2%)	4 (7.4%)	
	Extremely severe	2 (2%)	2 (4.3%)	0 (0%)	
	Mean (SD)	13.62 (8.65)	14.09 (9.87)	13.22 (7.54)	0.961^b^
DASS-A	No anxiety	65 (65%)	27 (58.7%)	38 (70.4%)	0.171 ^a^
	Mild	5 (5%)	2 (4.3%)	3 (5.6%)	
	Moderate	14 (14%)	6 (13%)	8 (14.8%)	
	Severe	11 (11%)	9 (19.6%)	2 (3.7%)	
	Extremely severe	5 (5%)	2 (4.3%)	3 (5.6%)	
	Mean (SD)	7.26 (6.56)	8.83 (7.09)	5.93 (5.81)	0,029^b^
DASS-D	No depression	61 (61%)	28 (60.9%)	33 (61.1%)	0.757^a^
	Mild	17 (17%)	9 (19.6%)	8 (14.8%)	
	Moderate	9 (9%)	4 (8.7%)	5 (9.3%)	
	Severe	10 (10%)	3 (6.5%)	7 (13%)	
	Extremely severe	3 (3%)	2 (4.3%)	1 (1.9%)	
	Mean (SD)	8.66 (8.61)	8.09 (8.73)	9.15 (8.55)	0.344^b^
DASS-Global	Mean (SD)	29.54 (20.81)	31.00 (22.79)	28.30 (19.08)	0.758^b^
EMCCoEnMu	Mean (SD)	3.09 (0.67)	3.06 (0.66)	3.12 (0.68)	0.665^b^
EMCProBas	Mean (SD)	2.47 (1.00)	2.54 (0.99)	2.42 (1.00)	0.697^b^
EMCAisSoc	Mean (SD)	3.11 (0.81)	2.30 (0.72)	2.94 (0.86)	0,024^b^
EMCTraIng	Mean (SD)	2.76 (1.09)	2.67 (1.13)	2.83 (1.06)	0.479^b^
EMCGlobal	Mean (SD)	2.94 (0.66)	2.95 (0.64)	2.93 (0.69)	0.841^b^

^a^χ-square; ^b^Mann-Whitney’s U test.

## DISCUSSION

This study showed a low level of psychological distress in general, in a sample of undergraduate student nurses who were about to begin their clinical placements during the academic year 2020-2021. Meanwhile, the fear of COVID-19 was moderate, specifically in relation to fear of infection, illness or death, and fear of social isolation. Furthermore, the student nurses did not think of themselves as vulnerable; regularly, their level of psychological distress was in relation to cohabiting with relatives or people who were perceived as being vulnerable to the infection.

Healthcare workers in general, and nurses in particular, have experienced higher levels of psychological distress during the COVID-19 pandemic than the general population. Specifically, in Spain, healthcare professionals experienced significant levels of stress, feelings of exhaustion and emotional overload, among other psychological symptoms^(19)^. Student nurses who were either working or on placement during the pandemic also experienced symptoms of psychological distress, including moderate to severe anxiety^(20)^.

The results from DASS-21 reveal that, in general, the student nurses’ level of psychological distress was relatively low before their clinical placement. However, it is important to highlight that, as opposed to previous studies in this population^(21)^, our data were collected in the last trimester of the year 2020, when more became known about the nature of the disease, and preventative measures, including personal protective equipment, became available and accessible to the students^(22)^. In addition, as the pandemic progressed, health systems became more competent in the management of the situation and new protocols were developed and implemented. For instance, some health services were organized in a double circuit for COVID-19 and non-COVID-19 patients^(23)^. This was done to reduce contact and prevent transmission between infected patients and non-infected patients and healthcare staff. Our undergraduate student nurses beginning their clinical placements in the autumn term were allocated to “COVID-19- free services” when possible, but this could not be guaranteed, especially in areas such as accident and emergency and community care.

Regarding the participants’ fear of COVID-19 before placement, the student nurses experienced a moderate level of fear, especially in the dimensions fear of infection, illness and death, and fear of social isolation. Similar results were obtained in a sample of healthcare students from Jordan^(14)^. Their fears were not unfounded as our undergraduate student nurses faced several challenges and difficulties directly and indirectly brought about by the pandemic. Namely, we identified a high level of anxiety in relation to social isolation. It is important to highlight that, up to the end of the summer 2020, the Spanish government’s main anti-COVID-19 strategy had been based on a countrywide lockdown first, from March to May 2020, and then on the confinement of specific groups, including those who were more vulnerable and those who had been in contact with a positive person or were positive themselves. Although strict lockdowns did reduce the incidence of the virus and avoid collapse of the Spanish health service, these measures had a negative emotional and psychological impact on the population^(24)^. However, this phenomenon was not exclusive to our country, but was replicated in other countries all over the world. For example, general lockdowns were found to be a contributing factor for increased stress^(11)^, anxiety and depression^(25)^ among college students during the pandemic.

As mentioned above, as well as social isolation, our participants scored higher in the dimension fear of infection, illness, and death, especially if they were living with relatives. Thus, we argue that the student nurses’ fear of becoming infected, falling ill, and even dying was not so much for themselves but for the possibility of infecting their significant others^(26)^. According to the International Council of Nurses^(13)^, over 1.6 million healthcare workers from 34 different countries had been infected with COVID-19. Furthermore, by this date, the cumulative number of reported COVID-19 deaths in nurses was 2262 in 59 countries. However, according to this same source, qualified nurses all over the world said that the risk of infecting others, and not themselves, was the greatest stress factor. This may explain why many healthcare workers, including nurses, put their relatives’ best interests first and left their homes and chose to live in isolation and away from their loved ones, especially at the beginning of the pandemic^(27)^. Yet, living in isolation often had a negative impact on the psychological status of these healthcare professionals. Although the student nurses on placement were, whenever possible, allocated to “COVID-free services”, they were in clinical areas and thus exposed to other professionals and patients who could be undiagnosed and/or asymptomatic, thus at risk of, unknowingly, transmitting the infection to their friends and relatives.

Generally, women outnumber men in the field of nursing; accordingly, our sample of student nurses was predominantly female. Although only a minority of male students took place in our study, we found no significant differences in the level of psychological distress between male and female student nurses. Similarly, several studies found equal rates of stress, anxiety and depression in male, female (or other) healthcare professionals and healthcare undergraduate students^(28)^. More specifically, according to previous reports^(29)^, mental distress experienced by medical undergraduate during COVID-19 was more severe than that of the general population, and the female students were more prone to develop depressive symptoms. In contrast, previous studies^(30)^ identified higher rates of anxiety, self-esteem, and irrational thinking in male students, contributing to a more impulsive and anxious response to stressful stimuli.

We found a significant association between the students’ living arrangement and fear of loss of employment and income. Not surprisingly, those who lived with their relatives, and were supported by them, were less worried about their financial situation than those who had alternative living arrangements. Previous investigations^(25)^ have detected an association between financial burden and psychological distress during mass quarantine periods. In fact, many students and their families have suffered loss of jobs and income during the pandemic^(24)^. Loss of employment and income may impact on the students and their families’ ability to pay academic fees and other costs associated to college life, which may result in students dropping out of university or delaying graduation. Unfortunately, it is not always easy for the students and/or the universities to find alternative ways of financing higher education studies.

Not knowing their upcoming placement location increased the students’ symptoms of anxiety. However, previous studies^(27)^ have identified an increase in the students’ symptoms of anxiety as they anticipate their clinical placement under normal circumstances. Thus, it is possible that this increase in the students’ symptoms of anxiety was unrelated to COVID-19. A follow-up study in a similar population once the situation is back to normal would be necessary to confirm this point. In contrast, knowing their upcoming placement location increased the students’ fear of social isolation. In our opinion, this may be due to the realization of the imminence of their clinical placement, which probably made the students reflect on the possible consequences of joining their qualified colleagues in the field. Upon reflection, it is possible that neither the faculty nor the clinical mentors address and manage clinical placement-related anxiety sufficiently under normal circumstances^(26)^. Student nurses need support before, during and after clinical placement, and both academic and clinical educators should be aware of this and systematically address these issues, even more so in these circumstances.

To our knowledge, this is the first study to measure psychosocial distress and fear of COVID-19 before clinical placement in Spanish student nurses during the pandemic. However, our findings should be interpreted with caution as there are some limitations to our study that we wish to highlight. First, our sample comprised student nurses registered at one single higher education institutions and, therefore, our results may not be applicable to the general population of Spanish student nurses. In addition, our findings allowed us to establish associations between some of the variables analyzed and psychological distress and fear of COVID-19 in our sample. However, a mixed methodological approach incorporating qualitative methods of data collections, such as personal interviews or focus groups, may have helped shed some light on the students’ feelings and emotions before commencing their clinical placement in the third trimester of the year 2021. Future studies should address this gap in the literature and minutely analyze and describe the student nurses’ pre-placement experience and needs, to inform the design of adequate strategies to support the students, both personally and academically, before, during and after placement.

## CONCLUSION

The student nurses who were living with relatives before commencing their clinical placement, experienced higher levels of stress than those who had other living arrangements. More symptoms of anxiety were associated with not knowing their upcoming placement location, whereas fear of isolation was associated with knowing their upcoming placement location. Additionally, the students who lived with their family were less fearful of loss of work and income than those who lived independently. We highly recommend that higher education institutions implement strategies and programs aiming at preventing and decreasing psychological distress among student nurses, not only under normal circumstances but especially during COVID-19 crisis.

## ASSOCIATE EDITOR

Thiago da Silva Domingos
