# ERRATUM: Psychological distress and fear of Covid-19 in student nurses before clinical placement: a cross-sectional study

**DOI:** 10.1590/1980-220X-REEUSP-2021-0548er-en

**Published:** 2022-06-22

In the article “Psychological distress and fear of Covid-19 in student nurses before clinical placement: a cross-sectional study”, with DOI code number: https://doi.org/10.1590/1980-220X-REEUSP-2021-0548en, published at Revista da Escola de Enfermagem da USP [online], v.56:e20210548, on the page 1:

**Where it was written:**

“Sofrimento psicológico e medo da Covid-19 em estudantes de enfermagem antes do estágio clínica: um estudo transversal”.

**Should read:**

“Sofrimento psicológico e medo da Covid-19 em estudantes de enfermagem antes do estágio **clínico:** um estudo transversal”.

